# Reclamation of wastewater in wetlands using reed plants and biochar

**DOI:** 10.1038/s41598-022-24078-9

**Published:** 2022-11-14

**Authors:** Amany A. Asaad, Ahmed M. El-Hawary, Mohamed H. H. Abbas, Ibrahim Mohamed, Ahmed A. Abdelhafez, Mohamed A. Bassouny

**Affiliations:** 1 Central Laboratory for Environmental Quality Monitoring, National Water Research Center, El-Qanater El-Khiria, Egypt; 2 Drainage Research Institute, National Water Research Center, El-Qanater El-Khiria, Egypt; 3 Soil and Water Department, Faculty of Agriculture, Benha University, Benha, Egypt; 4 Department of Soils and Water, Faculty of Agriculture, New Valley University, Kharga, Egypt; 5 National Committee of Soil Sciences, Academy of Scientific Research and Technology, Cairo, Egypt

**Keywords:** Environmental chemistry, Water microbiology

## Abstract

To cope with water crisis, wastewater reuse has been introduced as a potential source for irrigation. On the other hand, irrigation with wastewater may negatively affect the surroundings. In this study, reed plant (*Phragmits australis*) and its biochar were tested as low-cost treatments to enhance the efficiency of wastewater reclamation in wetlands within only 72 h. The investigated water was of low irrigation quality and exhibited high contents of BOD_5_ and fecal coliform. Moreover, this water contained high levels of soluble cations and anions; besides, being marginally contaminated with Cu, Mn and Cd. After 2 days in the sedimentation unit, wastewater was subjected to three reclamation treatments in parallel (each lasted for 24 h): (1) a “sand & gravel bed”, (2) “reed plants grown on a sand & gravel bed” and (3) “biochar + a sand & gravel bed”. The results showed that all treatments decreased BOD_5_, fecal coliform, total cations and anions, with superiority for the second and third treatments. The levels of the potentially toxic elements also decreased to values within the permissible levels. Although the aforementioned wastewater treatment processes upgraded the quality of this water, it remained in the poor grade. Biochar or reed plants grown on sand and gravel beds significantly improved wastewater quality to the medium quality grade, with superiority for biochar treatment. In conclusion, investigated treatments are guaranteed in wetlands for wastewater reclamation; yet, further protocols should be followed to achieve safe handling of this water and attain the sustainable goals.

## Introduction

Water scarcity is a critical issue threating the sustainability of many regions around the world^1,2^, especially in the Mediterranean African countries^3^. Egypt is one of these countries that use approximately 86% of the water resources^4^; nevertheless this amount is not enough to meet the demands of development^5^. Moreover, Egyptian farmers, who suffer from shortage in irrigation water, use waste water for crop production^6–8^ which has negative impacts on human health^9^. This crisis is thought to be worsen after the construction of the Grand Ethiopian Renaissance Dam^10^. Probably, the use of unconventional water resources has become an essential dispute to cope with water scarcity^11,12^; yet proper improvements in their quality should be taken into consideration^13–18^. In this context, the Egyptian government has constructed many plants to treat wastewater^19^, yet the daily amount of reclaimed water is still far below the intended needs.

The Egyptian Ministry of Housing, Utilities, and Urban Communities has set the latest code in 2015 to manage the use of treated wastewater for agricultural purposes^20^. According to this code, treated wastewater is classified into four categories based on the level of treatment, each devoted to irrigate particular crops. Although, this code regulates the usage of wastewater for crop production; however, the government could not offer suitable alternatives for the shortage of irrigation water in many arable lands. It is then thought that the improper management of this crisis may lead to food insecurity on one hand^21^ and threat the sustainability of land use in agriculture on the other hand^22^.The main risks associated with the usages of wastewater for irrigation are (1) their high contents of suspended and soluble organic matter, (2) the oversupply of nutrient loads and (3) their contents of potentially toxic elements (PTEs)^23^.

Constructed wetlands are probably the most common reclamation procedures used to lessen organic and inorganic pollutants in wastewaters as well as the pathogens^24^ using (1) abiotic mechanisms (such as sedimentation, filtration, chemical precipitation and adsorption) and (2) biotic mechanisms via organisms that contribute to reduce contaminant levels and/or remove them by vegetation uptake^25^. This approach is characterized by its simplicity, relative low cost, ease to operate and maintained versus the other traditional methods^26^.

In this study, four techniques were introduced to treat wastewater, within a relatively short period, using both biotic and abiotic wetland approaches. These approaches comply with the rules and regulations and have no direct or indirect negative impacts on the surroundings^27^. The first one is the sedimentation unit in which a primary treatment of wastewater occurs through precipitation of the suspended organic materials^28^. This may considerably lessen the bounded organic and inorganic contaminants^29^, even the fecal bacteria^30^. The second unit is the sand-gravel bed to attain an economic filtration of wastewater^31^. This treatment may further lessen the organic material; hence decrease COD, BOD_5_ beside of diminishing the existence of fecal bacteria. The third unit contained common reed plants grown on a sand bed. This plant is an aquatic one that is used in constructed wetlands for wastewater reclamation^32^. It can effectively minimizes the potentially toxic elements^33^; total dissolved solids (TDS), numbers of bacteria^34^, BOD_5_, COD, ammonium and phosphate in domestic wastewater^35^.

The fourth unit contained biochar derived from common reed plants mixed with sand. In this aspect, biochar is a carbonaceous material produced through thermal pyrolysis of organic wastes in oxygen-deficit conditions^36–38^. This product is of high porous structure besides being rich in functional groups^39^. Accordingly, biochar is widely used for removal of potentially toxic elements^40^ and nutrients in wetlands^41,42^. It may also lessen considerably COD, concentrations of ammonia^43^, organic and inorganic contaminants^44^. Although, active carbon is widely used as an efficient adsorbent to remove organic pollutants from aqueous solutions^45^ such as dyes^46^ and is used also for immobilizing microorganisms when being used as a thick biofilm coating the filler surface^47^; yet biochar is more favorable than activated carbon, because biochar costs less beside of its ability to sequester carbon^45^.

The current study investigates the effectiveness of using reed plant *(Phragmits australis*) versus its biochar to enhance the efficiency of wetlands for wastewater reclamation within only 72 h. The efficiency of using this type of biochar to attain this aim is not so far investigated and this in-situ rapid and low-cost effective technique may be applicable to reclaim large amounts of wastewaters within short time periods in order to avoid their negative implications on the surrounding environment. Water quality parameters were then estimated before and after treatments then the after-treatment values were included in a model (Irrigation water quality index, IWQI) which was modified by Jahin et al.^48^ to estimate the overall efficiency of each unit in improving water quality.

Specifically, we anticipate that both biotic (reed plants) and abiotic (reed biochar) options may effectively be used for enhancing the treatment efficiency of 100L of wastewaters in only 72 h (Hypothesis 1); yet, the abiotic approach could be more preferable than the biotic one in wastewater reclamation because of the high selectivity of the grown plants to absorb contaminants from wastewater (Hypothesis 2). This study is; therefore, of high priority to improve the quality of marginal waters, worldwide, to meet the demands of development using safe, low cost small portable units and is therefore considered an important goal in the Egyptian vision 2030.

## Materials and methods

### Materials of study

Wastewater samples were collected from Bahr Elbaqr drain, whose coordinates are 31° 9′ 49.82″ N, 32° 11′ 54.43″ E, at four respective dates i.e., 29/8/2019, 2/9/2019, 10/9/2019 and 25/11/2019. A point to note is that this drain receives treated and untreated domestic and industrial sewages. These samples were mixed together to make a composite sample whose chemical characteristics are presented in Supplementary Table 1. Further analyses are mentioned in the “Results and discussion” section (marked with the symbol T_0_).

The investigated wastewater is of high salinity (EC > 3 dSm^−1^) and also exhibits Mg hazards (Mg ratio > 50%). Plantlets of reed (*Phragmits australis*) were obtained from the nearby areas of fresh water canals. Biochar was prepared from reed plants through slow pyrolysis of biomass in a controlled unit for 1 h at 450 °C. This temperature is enough to produce a high biochar yield of big stability^49^. On the other hand, acidifying this biochar could increase its surface area^50^ to adsorb more contaminants from wastewaters. Accordingly, our C-rich product was then acidified according to Liu et al.^51^ by immersing it in H_2_SO_4_ (concentrated); then washed with distilled water several times to get rid of excess acidity; afterwards, acidified biochar was dried at 105 °C. Its chemical properties are presented in Supplementary Table 2.

### Characteristics of biochar

#### FTIR

The functional groups of biochar was analyzed in the Department of chemistry, science faculty, Suez Canal University via Fourier transform infrared spectrophotometer (Bruker Tensor37, Billerica, MA, USA), using KBr discs within the wavenumber range of 4000–400 cm^−1^ and a resolution of 2 cm^−1^.

#### Scanning electron microscopy (SEM)

Scanning electron microscope (ZEISS, Japan) of 7 kV voltage was used for the surface observations of biochar, while 15 kV voltage were used for identifying the deeper structure. The elementary composition of biochar was detected using an X-ray energy dispersive spectrometer EDX (20 mm^2^ SDD detector) and also by ZEISS. These analyses were conducted in the Egyptian petroleum research institute (EPRI).

#### X-ray diffraction (XRD)

XRD was measured in the Egyptian Petroleum Research Institute (EPRI) using an advanced diffractometer (PAN analytical XPERT-PRO) equipped with a Cu-K radiation source (X = 0.154 nm) at room temperaturet and data was collected within the range of [°2θ] 4°–60° using a fixed time mode with a step interval of 0.02. These patterns were progressed with Ni-filtered copper radiation (λ = 1.54060 Å) at 40 kV and 40 mA.

### Experimental design

Wastewater was pumped into a plastic container of one cubic meter capacity (sedimentation unit, T_1_) and left for 48 h. Thereafter, this wastewater was re-distributed on three rectangular tanks (each was replicated 3 times) through a perforated acrylic pipe, with an inside diameter 1.25 cm. These tanks (cells) were made of glass with internal dimensions of 1.2 m length, 0.4 m width, and 0.5 m depth (For more details, see Supplementary Table 3). Each cell was then packed with a layer of gravels (20 cm) in its bottom above which sand was applied uniformly to maintain a layer of 20 cm thick. A plastic mash was used to separate between the two layers in each cell.

**Figure 1:**
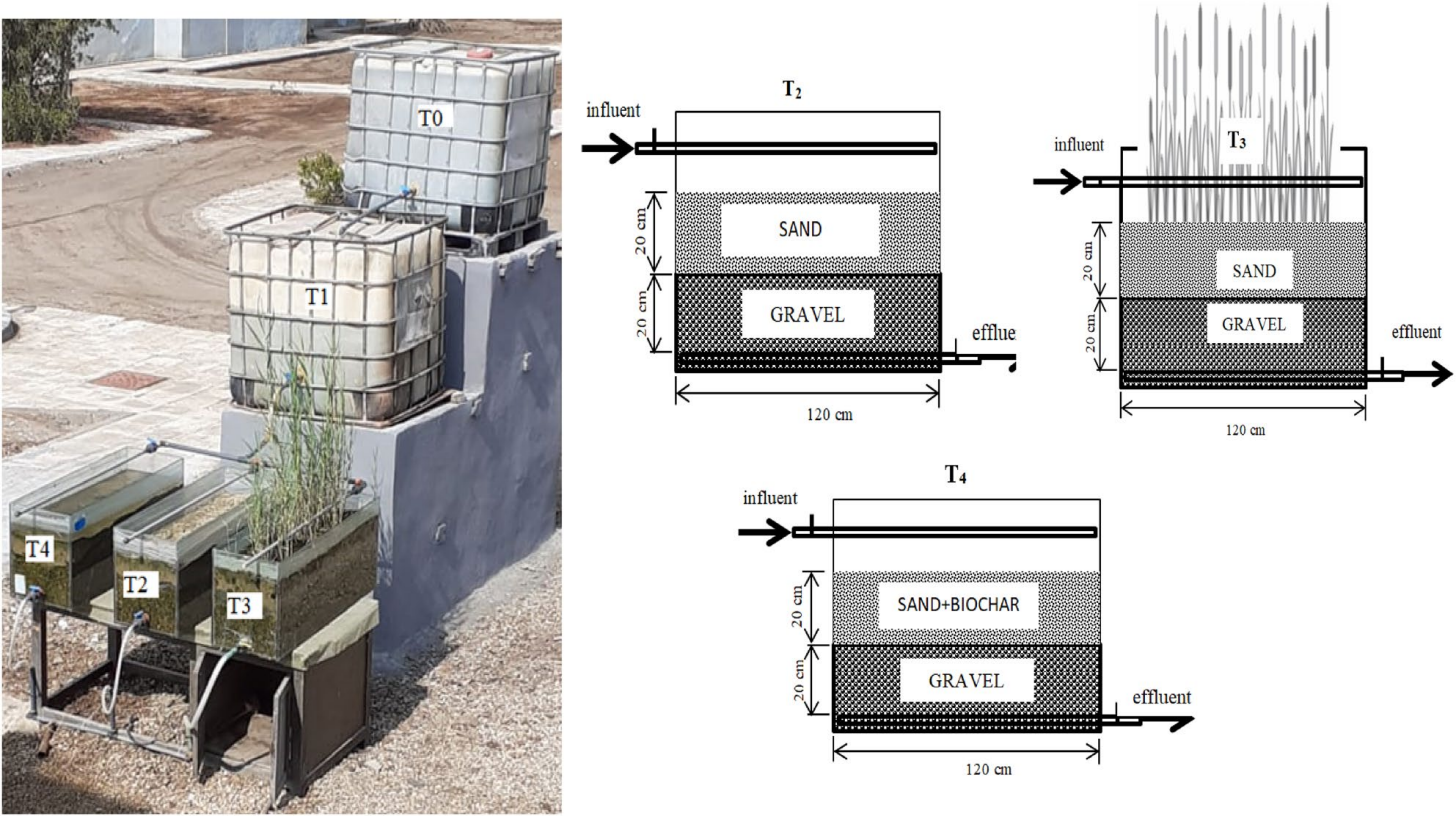
Schematic diagrams of the treatment cells using sand and gravel beds (T_2_) grown with common reed plants (T_3_) and mixed with biochar (T_4_).

The first tank contained only sand gravel beds without further additions (a reference cell, T_2_), while the second one was planted with four seedlings of common reed (*Phragmites australis*), of 1 year old transplanted from the areas nearby fresh water canals (T_3_), whereas the third tank contained biochar mixed with the sand layer at a rate of 2 kg m^−3^, T_4_ (Fig. 1). All tanks have inlet valves quite above the sand layer (connected to an air vent pipe) to receive the wastewater from the sedimentation unit and also contained two parallel acrylic perforated pipes, placed at the bottom of each tank, to collect the effluents to the outlets. These outlets were very close to the bottom to allow steady downward follow of the treated water through these tanks at a rate of 1.15 × 10^−6^ m3 s^−1^ ; hence, achieve an overall discharge of approximately 100 L of treated wastewater per day).

### Water analyses

Water analyses were conducted within 24 h after collection in the “Central Laboratory of Environmental Quality Monitoring (CLEQM), National Water Research Center (NWRC), Cairo, Egypt” (a certified laboratory, ISO 17,025: 2005). Na^+^ and K^+^ concentrations were measured in water samples by flame photometer (Sherwood model-410, England), Ca^2+^, Mg^2+^, CO_3_^2−^ and HCO_3_^−^, Cl^−^ ions were determined by titration according to APHA^52^, while NO_3_^−^, SO_4_^2−^ and PO_4_^3−^ were estimated using ion chromatography system (ICs5000-Dionex, USA). Potentially toxic elements (PTEs) were determined using Inductively Coupled Plasma-Optical Emission Spectrophotometer (ICP-OES, Perkin Elmer Optima 5300, USA). Chemical oxygen demand (COD) was determined as outlined by APHA^52^ based on the Colorimetric Method 5220 D Closed Reflux, while the biochemical oxygen demand (BOD_5_) was determined according to the same reference but for 5 days using Method 5210 B. Total and fecal coliform counts were also estimated according to 9221 B and 9221 E methods, respectively^52^.

### Quality control measures

All chemicals used in this study were of analytical grade (obtained from Merck Company, Germany). Glassware were left overnight in dilute nitric acid (10%) before use, and then washed thoroughly with deionized water. Blank and reagents were prepared using double deionized water (Milli-Q, Millipore; < 18 MΩ cm at 25 °C) to ensure accuracy. Spikes were also considered for PTEs determinations by ICP instrument and the recovery values were acceptable (within 91 ± 3%). Portions of the used biochar and sand that was used in wetlands underwent acid digestion with aqua regia method and their contents of PTEs were determined by Inductively Coupled Plasma-Optical Emission Spectrophotometer (ICP-OES, Perkin Elmer Optima 5300, USA).

### Data analyses

The obtained data were statistically analyzed using SPSS statistical software (ver 18) through the analyses of variance (one-way ANOVA) and Dunken’s test to signify significant variations among means (*P* < 0.05). All figures were plotted using Sigma Plot 10.0 Software. Water quality indices were estimated according to FAO^53^ as follows:

1 $${\text{Sodium adsorption ratio}}\quad \left( {{\text{SAR}}} \right) = \frac{{{\text{Na}}^{ + } /23}}{{\sqrt {\frac{{({\text{Ca}}^{2 + } /20) + ({\text{Mg}}^{2 + } /12)}}{2}} }}$$

2 $${\text{Residual sodium carbonate}}\quad \left( {{\text{RSC}}} \right) = {\text{CO}}_{3}^{2 - } /30 + {\text{HCO}}_{3}^{ - } /61) - \left( {{\text{Ca}}^{2 + } /20 + {\text{Mg}}^{2 + } /12} \right)$$

3 $${\text{Mg}} - {\text{ratio}} = \frac{{{\text{Mg}}^{2 + } /12}}{{\frac{{{\text{Mg}}^{2 + } }}{12} + {\text{Ca}}^{2 + } /20}} \times 100$$

Note: all calculations were conducted on bases of ion concentrations expressed in mg L^−1^. Irrigation water quality index (IWQI) was then calculated according to the model of Jahin et al.^48^

4 $${\text{IWQI}} = { }\mathop \sum \limits_{{{\text{i}} = 0}}^{{\text{n}}} {\text{S}}_{{\text{i}}} \times {\text{W}}_{{\text{i}}}$$

where S_i_ is the unit-less score of a single index (ranges from 0 to 100%) calculated from the following equation:

5 $${\text{S}}_{{\text{i}}} = \left[ {1 - \left( {\frac{{{\text{Va}} - {\text{Vi}}}}{{{\text{Vs}} - {\text{Vi}}}}} \right)} \right] \times 100$$

V_a_ and V_s_ are the measured and reference values (introduced by FAO). V_i_ is the ideal value of each parameter which is estimated by zero for all parameters except pH (valued 7).

## Results and discussion

### Characterization of biochar

#### XRD for biochar

**Figure 2:**
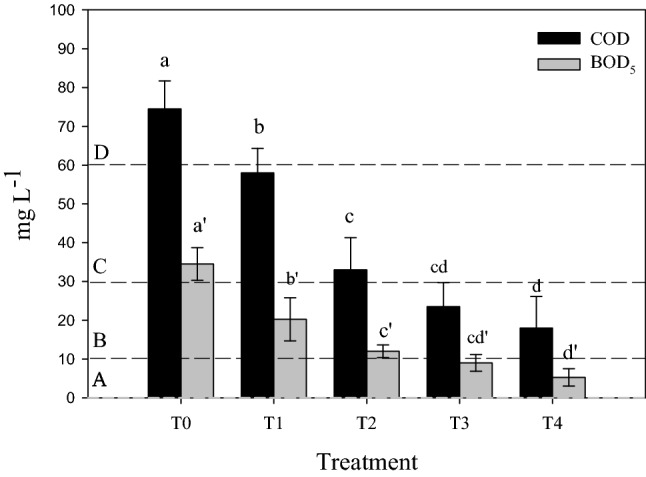
COD and BOD5 in wastewater collected from each treatment unit (the dashed-lines presented the different categories of BOD5 according to the Egyptian Code). T0 (untreated water), T1 (water after sedimentation unit), T2 (water after sand & gravel beds), T3 (water after reed + sand & gravel beds) and T4 (water after biochar + sand & gravel beds). Similar letters indicate no significant variations among treatments.

No distinctive sharp peaks was notices on the XRD pattern of biochar (supplimentary Fig. 2A) and the broadbands of this biochar appeared at 20–30 O within the 2θ range. This might indicate that biochar was of amorphos structure.

### FTIR spectrum

The OH groups was identified with strong and broad peaks between 3600 and 3000 cm^−1^, while the C–H stretching vibration appeared between 3000 and 2800 cm^−1^ (Fig. 2B). Also, the stretching vibration of C=O bonds was noticed between 1800 and 1600 cm^−1^ while the C=C vibrations of the aromatic rings was shown between 1600 and 1500 cm^−1^. This indicates that the used biochar was rich in the functional groups.

#### SEM-EDX

This analysis was performed to show the morphology of biochar surfaces before and after exposure to wastewater. Generally, biochar was of porus structure and its particles were within the nanoscale. This might indicate its high specific area. The SEX-EDX analysis showed the high adsorption rate of biochar on the surface.

### Chemical and biological oxygen demands

Chemical and biological oxygen demands are two important indices commonly used to evaluate the organic loads in water^54^. Results obtained herein indicate that COD contents in all water samples did not exceed the permissible level presented by FAO which is 250 mg L^−1^, even in the raw wastewater of Bahr Elbaqr. The other parameter that is used to evaluate the level of water pollution with the organic contaminants is the biological oxygen demand (BOD_5_). Its level was relatively high in the raw waste water (T_0_) of Bahr Elbaqr and exceeded the permissible level which is only ≤ 20 mg L^−1^ for vegetables that can be eaten uncooked^55^. It falls within the third category of the Egyptian Code^20^ for using wastewater in irrigation and this indicates that this water is not suitable for irrigating fruit trees, cooked and processed vegetables, dry cereal crops and medical plants. Generally, the organics are brought mainly to water bodies via anthropogenic activities^56^. Higher values of either chemical or biological oxygen demands indicate low water quality^57^.

Sedimentation process in the precipitation unit significantly decreased the values of both COD and BOD_5_. Further reductions in these two parameters occurred when the studied wastewater passed through “sand and gravels beds” where these beds acted as natural filters that retained the organic materials suspended in water^58^. Further reductions in values of both COD and BOD_5_ occurred when beds were planted with reed plants (T_3_) or mixed with biochar (T_4_), with superiority for the biochar treatment (Fig. 2). In this context, biochar removed partially the suspended and dissolved organic materials from water via electrostatic attraction on its heterogeneous surfaces^59^; besides it might induce the microbial activities to degrade the organic contaminants in water^60^.

In case of reed-plants, they probably minimized the activities of anoxic-anaerobic microorganisms; thus diminished both COD and BOD_5_ considerably^61^. Similar results indicate that this plant type reduced effectively both COD and BOD in the polluted water of river Ravi, Pakistan by 85.9 and 83.3%, respectively within only 96 h^62^. Likewise, Yasar et al.^63^ found that reed plants considerably lessened COD and BOD by 71.90 and 64.29%, respectively. Overall, these two treatments improved considerably water quality to become within category “A” (based on its content of BOD_5_ i.e. < 10 mg L^−1^) according to the Egyptian code.

### Total and fecal coliform concentrations

Coliform bacteria are considered indicators of fecal contamination in water^64^. The permissible level is below 1000 CFU/100 mL^65^. In the current study, total counts of coliform exceeded 50, 000 CFU/100 mL in the raw wastewater of Bahr Elbaqr. This extremely high value was comparable with the one recorded by Elbaha et al.^66^ in wastewater sampled from Bahr El-Baqr drain whose total count of coliform exceeded 40,000 CFU/100 mL. Probably the presence of the high loads of organic matter in water might account for such increases^67^. In particular, fecal coliform bacteria are of special concern because these bacteria are originated mainly in the intestinal gut of warm-blooded animals^67^. Thus, their counts are preferably used rather than the total counts of coliform for evaluating the risk assessment of microbial pollution in water^68^.

**Figure 3:**
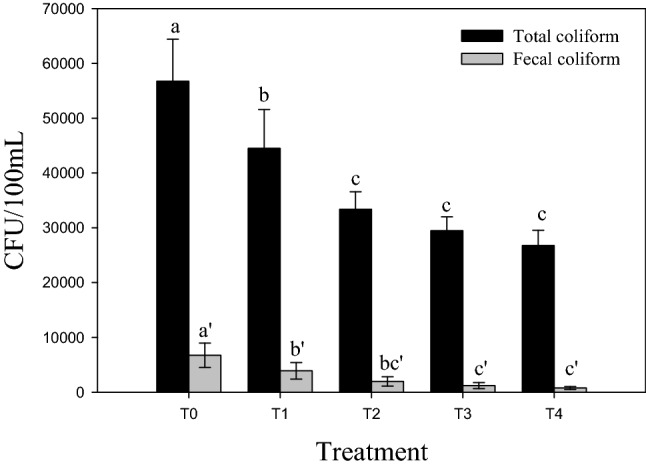
Total and fecal coliform in wastewater collected from each treatment unit. T_0_ (untreated water), T1 (water after sedimentation unit), T_2_ (water after sand & gravel beds), T_3_ (water after reed + sand & gravel beds) and T_4_ (water after biochar + sand & gravel beds). Similar letters indicate no significant variations among treatments.

Results obtained herein (Fig. 3) indicate that the level of fecal coliform was above the acceptable level for irrigation water presented by FAO which is ≤ 1000 cells per 100 mL^53^, and also falls within category “D” according to the Egyptian code of the treated municipal wastewater (its log value was higher than 3.7). On the other hand, these counts decreased noticeably owing to the different treatment strategies. In this concern, the sedimentation unit lessened significantly total and fecal counts of coliform; yet, the primary treatment solely could not remove considerable levels of fecal coliform from wastewater^69^.

Passing wastewater through “sand & gravel” beds caused further reductions in the suspended organic materials; and this might in turn decrease significantly the fecal coliform counts in water. Similar results indicate that FC counts decreased by 69.38% in sand/gravel beds^70^. A point to note is that passing wastewater through “biochar + sand beds” T_3_ or sand cultivated with reed plants (T_4_) recorded additional reductions in both total and fecal counts of coliform, because biochar lessens microbial propagation^71^, while reed plants probably release toxins for pathogen disinfection^72^. Nevertheless, the log CFU /100 mL was still within Grade “D” after the aforementioned treatments, i.e., T_3_ and T4. Probably, the incubation period was not long enough to attain successful decontamination of wastewater from total and fecal coliforms; hence, further protocols should be considered to minimize these counts in wastewater.

### The pH of the wastewater

**Figure 4:**
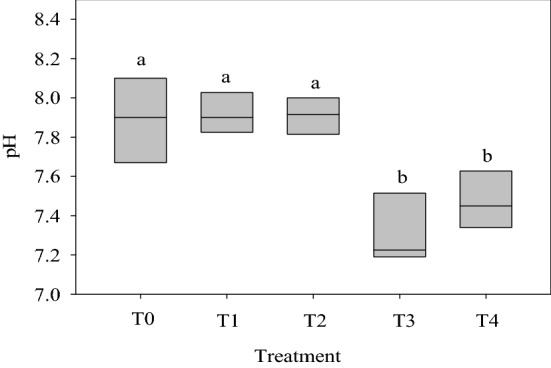
pH of the wastewater collected from each treatment unit. T_0_ (untreated water), T1 (water after sedimentation unit), T_2_ (water after sand & gravel beds), T_3_ (water after reed + sand & gravel beds) and T_4_ (water after biochar + sand & gravel beds). Similar letters indicate no significant variations among treatments.

The raw wastewater of Bahr El-baqr is of alkaline nature (its pH ranges from 7.6 to 8.1, Fig. 4). These values are within the acceptable ones of FAO^53^. Settlement of this wastewater in a sedimentation unit and/or passing it on sand and gravel beds did not affect significantly the pH of this water. However, in presence of either reed plants or biochar mixed with “sand and gravel” beds, the pH of wastewater decreased noticeably. This occurred in spite of the alkaline nature of the used biochar^38^; yet the functional groups of biochar probably buffer the pH of this water^73^ via deprotonating the negative sites on carbon surfaces^74^ which could increase CEC of the biochar and hence became able to retain more basic cations on its surfaces^75^. In case of *P. australis*, this plant might release acidic root exudates which, in turn, decreased the pH of the wastewater^76^. Similar results were reported by Shahid et al.^62^ who found that reed plants decreased noticeably the pH of the polluted water of river Ravi, Pakistan from 8.5 to 8.37 and 7.73 within only 24 and 96 h^62^.

### Soluble cations and anions in water

Results obtained herein indicate that the dominant cation in wastewater is Na^+^ while the dominant anion is Cl^−^ (Table 1). Concentrations of both elements exceeded the permissible levels of FAO (920 mg L^−1^ for Na^+^, 1065 mg L^−1^ for Cl^−^)^53^; accordingly, irrigation with this water may exhibit the symptoms of sodium and chloride toxicity on plants. Likewise, Mg^2+^ concentration surpassed the acceptable level in raw wastewater (60 mg L^−1^), while Ca^2+^, HCO_3_^−^, N-NO_3_^−^, N-NH_4_^+^, PO_4_^3−^ and SO_4_^2−^ were within the permissible levels for irrigation (400 mg L^−1^ for Ca^2+^, 610 mg L^−1^ for HCO_3_^−^, 10 mg L^−1^ for NO_3_^−^, 5 mg L^−1^ for NH_4_^+^, 2 mg L^−1^ for PO_4_^3−^ and 960 mg L^−1^ for SO_4_^2−^). In this concern, high concentrations of N-NO_3_^−^, and orthophosphate are indicators of water pollution due to anthropogenic activities^56^. The wastewater of Bahr Elbaqr drain also recorded not detectable concentrations of CO_3_^2−^ ions (0 mg L^−1^).

**Table 1:** Concentrations of soluble cations and anions in wastewater collected from each treatment unit.

	T_0_	T_1_	T_2_	T_3_	T_4_	FAO limit
Ca^2+^	142.0^a^ ± 7.7	115.0^b^ ± 5.8	108.0^b^ ± 4.3	100.0^c^ ± 4.8	90.0^d^ ± 3.8	400
K^+^	33.6^a^ ± 1.4	32.7^a^ ± 1.2	24.4^b^ ± 1.4	23.8^b^ ± 1.1	23.4^b^ ± 0.9	5
Mg^2+^	121.3^a^ ± 8	120.4^a^ ± 7.1	85. 7^b^ ± 4.5	83.5^b^ ± 5.1	92.4^b^ ± 5.0	60
Na^+^	829.0^a^ ± 41.8	820.0^a^ ± 38.7	612.0^c^ ± 28.6	558.0^c^ ± 29.7	736.0^b^ ± 26.8	920
NH_4_^+^	2.8^a^ ± 0.17	2.1^b^ ± 0.16	1.4^c^ ± 0.08	0.1^e^ ± 0.01	1.1^d^ ± 0.05	5
HCO_3_^−^	488.0^a^ ± 20	440.0^b^ ± 20	439.0^b^ ± 15	414.0^bc^ ± 8	400.0^c^ ± 9	610
Cl^−^	1092.0^a^ ± 52	1090.0^a^ ± 51	1123.0^a^ ± 48	1056.2^a^ ± 32	1060.9^a^ ± 29	1065
NO_3_^−^	1.8^a^ ± 0.06	1. 8^a^ ± 0.08	1.8^a^ ± 0.07	0.2^c^ ± 0.01	1.2^b^ ± 0.08	10
Total- PO_4_^3−^	1.3^a^ ± 0.15	0.8^b^ ± 0.1	0.6^c^ ± 0.07	0.2^d^ ± 0.008	0.2^d^ ± 0.04	2
SO_4_^2−^	319.7^a^ ± 18.9	293.4^bc^ ± 17.6	288.9^c^ ± 15.8	309.4^ab^ ± 16.7	221.4^d^ ± 13.5	960

T_0_ (untreated water), T1 (water after sedimentation unit), T_2_ (water after sand & gravel beds), T_3_ (water after reed + sand & gravel beds) and T_4_ (water after biochar + sand & gravel beds). Similar letters indicate no significant variations among treatments. Mean with the same letter within rows are not significantly different at *P* < 0.05.

Treating wastewater via sand-gravel beds (plus reed plants or biochar) lessened significantly concentrations of Ca^2+^, K^+^ and Mg^2+^ in water with no significant variations among T_2_, T_3_ and T_4_ treatments. Moreover, these three treatments decreased significantly the concentrations of NH_4_^+^, HCO_3_^−^, and total PO_4_^3−^; and, the superiority in this concern was recorded for the T_3_ treatment (reed plant grown on a sand-gravel bed). In this context, sand beds could effectively decrease concentrations of ammonia and oxidized N-species in water by 30.4%^70^. Additionally, reed plant is a heavy feeder for N^72,77^; thus T_3_ treatment lessened considerably N-content in wastewater. These results are similar, to some extent, with those of Shahid et al.^62^ who found that *P. australis* significantly lessened the concentrations of NO_3_^−^ (from 33.3 to 24.13 mg L^−1^), total N (from 37.50 to 26.37 mg L^−1^) and P (from 26.3 to 2.1 mg L^−1^) in the polluted water of the river Ravi (Pakistan).

Biochar also proved its efficiency in reducing concentrations of N–NH_4_^+^, N–NO_3_^−^ and total- PO_4_^3−^ in wastewater versus T_2_. Similar results indicate that biochar as a filter media in column experiments reduced the concentrations of N-NO_3_^−^ and total- PO_4_^3−^ by 86% and 47%, respectively^78^. This might take place through retaining the ions on biochar via different mechanisms e.g., surface adsorption, chemical bonding and van der Waals force^79^. In case of SO_4_^2−^ ions, the highest reductions occurred due to T_4_, then T_2_ and T_1_.

### Potentially toxic elements (PTEs) in water

Potentially toxic elements (PTEs) are among the main threats that affect negatively the quality of water for drinking and irrigation purposes^37^. These contaminants do not undergo biodegradation^80^, accumulate in soil upon usage for irrigation^81,82^ and find their way to the food chain; hence, possess high potential health treats to man and animal^83^. Tracking the levels of any potential contaminant in water is necessary to control and prevent further environmental pollution^84^. Thus, the collected wastewater samples of Bahr Elbaqr were analyzed for its contents of PTEs (Supplementary Table 4), and the results obtained herein indicate that this water was marginally contaminated with Zn, Cu and Cd while the concentrations of the other PTEs did not exceed the permissible levels. Furthermore, they were found in low concentrations and some of them were below the detected limits of the measuring devise.

Reclamation of such a contaminated water should be considered to alleviate water resource crisis^85^. Three metal ions i.e. Zn, Cu and Cd were monitored prior, during and after reclamation in the current study. Also, Al concentrations were tracked during water reclamation, while the other PTEs were very low and almost below the limit of detection of the ICP instrument. A point to note is that total concentrations of the investigated PTEs i.e. Zn, Cu, Cd and Al in sandy soil were 210, 130, 1.2 and 17.1 mg kg^−1^, respectively while the corresponding concentrations in biochar after treatment were 672, 103, 8.6 and 784 mg kg^−1^, respectively. As a result, the removal mechanism might be attributed to the surface adsorption and chemical bindings with functional groups of the used biochar as indicated by the SEM–EDX analysis with the precipitation of metal ions on the surface of biochar particles.

**Figure 5:**
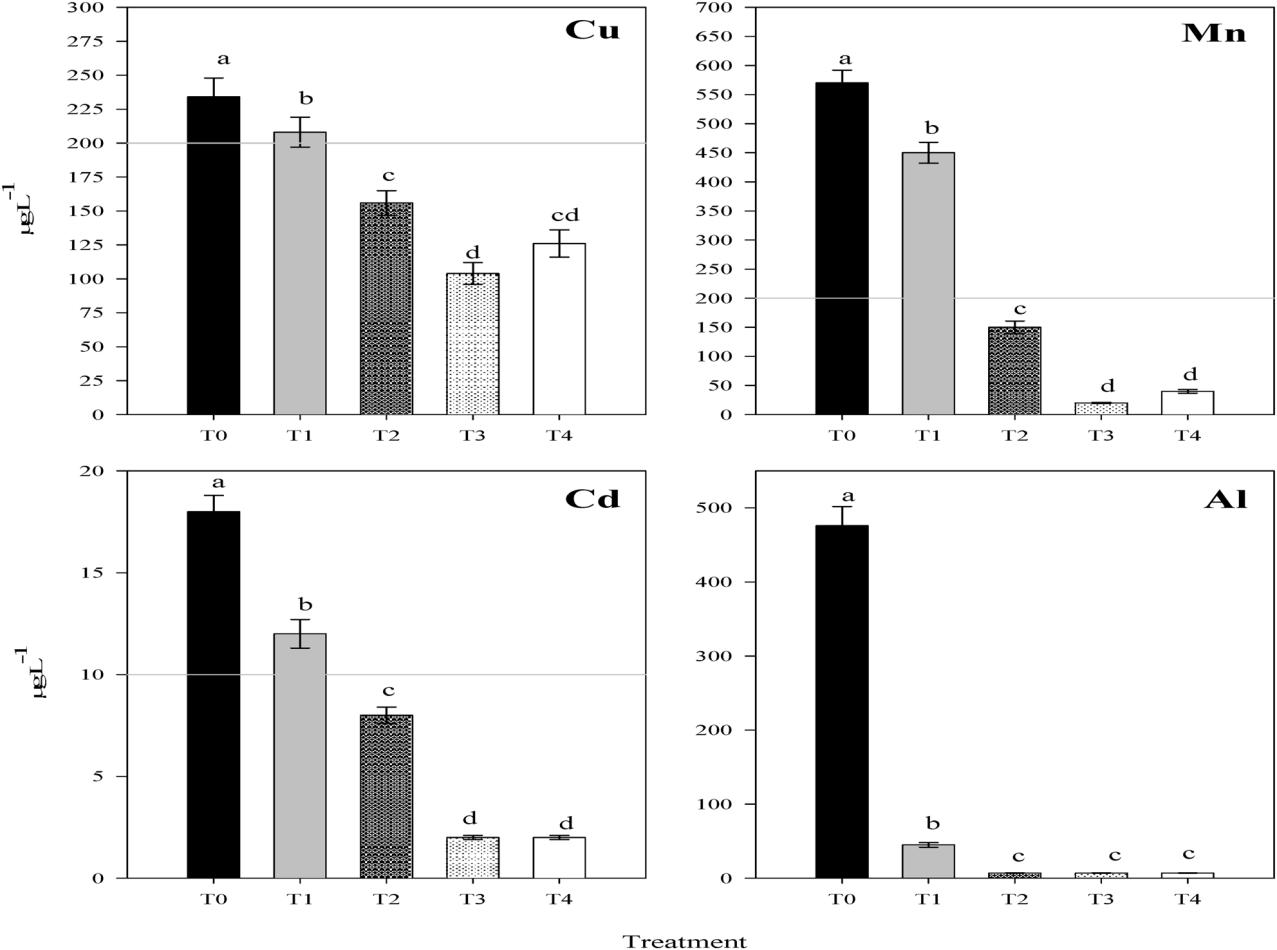
Concentrations of Cu, Mn, Cd and Al in in wastewater collected from each treatment unit (FAO permissible levels were presented by the gray lines, i.e. 200 µg Cu L^−1^, 200 µg Mn L^−1^, 10 µg Cd L^−1^ while in Al all values were below permissible level: 5 mg Al L^−1^). T_0_ (untreated water), T1 (water after sedimentation unit), T_2_ (water after sand & gravel beds), T_3_ (water after reed + sand & gravel beds) and T_4_ (water after biochar + sand & gravel beds). Similar letters indicate no significant variations among treatments.

Significant reductions occurred in concentrations of the investigated PTEs in wastewater due to the different reclamation treatments (Fig. 5). In this concern, the sedimentation unit (primary treatment) lessened considerably the concentrations of Mn^2+^, Cu^2+^, Cd^2+^ and Al^3+^ versus their concentrations in raw wastewater. Probably, precipitation of the suspended materials, which act as carriers of PTEs^29^ took place during this process^86^; thus, PTEs concentrations decreased considerably in wastewater^29^. In “sand-gravel” beds, supplementary reductions occurred in their concentrations.

In case of the reed and biochar treatments, additional reductions occurred in concentrations of Cd^2+^, Cu^2+^ and Mn^2+^ while these two treatments seemed to be ineffective in reducing the concentrations of Al^3+^ in wastewater beyond the concentrations achieved due to water filtration through “sand + gravel” beds only (T2). The efficiency of biochar to remove potentially toxic elements (PTEs) from wastewater was well investigated^87^. These metal ions were probably complexed with –OH and –COOH groups on biochar surfaces and also via electrostatic interaction with O-containing surface functional groups^88^. Other mechanisms might exist such as reduction, electron shuttling, and physisorption^89^. Accordingly, biochar effectively lessened the concentrations of Mn^2+^^90^, Cd^2+^ and Cu^2+^^88^ in water. In this context, it was found that biochar could effectively lessen the concentrations of soluble Cd in water within 24 h by approximately 95%^91^ and these results were a little bit higher than the findings obtained herein. Also, Reddy^78^ found that biochar could effectively decrease Cu concentrations from aqueous solutions by 65% when used as a filter media in column experiments.

In case of reed plants, these plants are characterized by their effective antioxidative metabolism in presence of high concentrations of metal contaminants^92^. Accordingly, these plants function as phytostabilization for Zn^2+^ and Cu^2+^^93^ while exhibit high absorption capacity for Cd^2+^^94^. Similar results indicate that the removal efficiencies of Cu and Cd from aqueous solutions by *Phragmits australis* were 50.8 and 42.2%, respectively^92^.

### Irrigation water quality index (IWQI)

**Figure 6:**
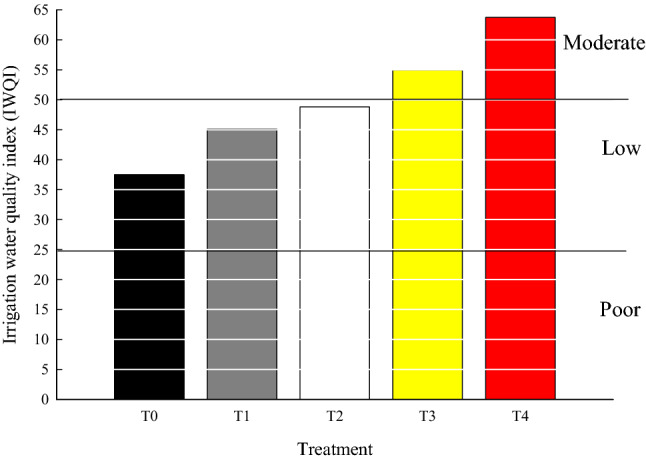
Irrigation water quality index calculated for wastewater collected from each treatment unit. T_0_ (untreated water), T1 (water after sedimentation unit), T_2_ (water after sand & gravel beds), T_3_ (water after reed + sand & gravel beds) and T_4_ (water after biochar + sand & gravel beds). Similar letters indicate no significant variations among treatments.

An overview on the quality of water was considered based on the calculations of the model introduced by Jahin et al.^48^ to quantify the success of the wastewater reclamation units for achieving sustainable environmental goals. This model put scores on the different indexes of irrigation water, assign a weight for each parameter, then calculate the final score from 0 to 100. In the current study, we used the following parameters to assess the quality of treated wastewater for irrigation purposes i.e. COD, BOD_5_, fecal coliform, water pH, soluble cations and anions as well as the four PTEs which exhibited significant variations among treatments (Cu^2+^, Mn^2+^, Cd^2+^ and Al^3+^). Components, with eigenvalues more than one, were considered presenting 74.40% of the variance of data for irrigation according to IWQI. Based on Jahin’s et al.^48^ classification, water quality is classified into five categories i.e. excellent (91–100%), good (71–90%), moderate (51–70%), low (26–50%), and poor (0–25%). The raw wastewater of Bahr Elbaqr drain could be classified as water of low grade (Fig. 6). T_1_ and T_2_ treatments improved the quality of water; yet it was still within the low quality grade (< 50%). T_3_ and T_4_ improved considerably wastewater quality up to be within the medium quality grade (55 and 63.75%, respectively), with superiority for T_4_ treatment. Based on the above results, both biotic (reed plants) and abiotic (reed biochar) approaches seemed to be effective in wastewater reclamation within only 72 h and therefore these findings supported the first hypothesis yet, more treatments are still needed to upgrade the quality of water to reach more environmentally desirable levels. Moreover, the superiority of T_4_ (abiotic) versus T_3_ (biotic) treatment in improving the quality of water verified the second hypothesis.

## Conclusion

Treating wastewaters has become an obligation to achieve the developmental goals of sustainability. In this study, reed plants and its biochar were investigated for their efficiencies to improve the performance of wetlands used for wastewaters reclamation. Within only 72 h, these two treatments reduced significantly fecal coliform counts, the levels of COD and BOD_5_, concentrations of major cations and anions as well as the potentially toxic element (Cu^2+^, Mn^2+^, Cd^2+^ and Al^3+^) contents in wastewater. The after-treatment values were included in a model (Irrigation water quality index, IWQI) to estimate the overall efficiency of these two treatment in improving water quality and the results indicated that the quality of wastewater was upgraded from low to medium class with superiority for the biochar treatment. Nevertheless, the obtained IWQI values (55–63.75%) were still lower than the expected ones; so, further treatments are needed to attain more environmentally desirable levels of contaminants in wastewater. Also, desorption mechanisms of contaminants from this type of biochar should be considered in further investigations.

## Supplementary Information

Supplementary Information.

## Data Availability

The datasets generated during and/or analyzed during the current study are available from the corresponding author on reasonable request.
