# Supplementary material for: Reclamation of wastewater in wetlands using reed plants and biochar
Source: Sci Rep. 2022 Nov 14;12:19516. doi: 10.1038/s41598-022-24078-9 (PMC9663436; doi:10.1038/s41598-022-24078-9)
Supplement: Supplementary file 1 — Supplementary Information. [file 41598_2022_24078_MOESM1_ESM.docx]

**Supplementary Table** **1.** Chemical characteristics of the investigated wastewater

| Parameter | pH | EC | SAR | RSC | Mg ratio |
| --- | --- | --- | --- | --- | --- |
| Value |  | dS m^-1^ |  | mmol_c_ L^-1^ | % |
| Estimated value | 7.89±0.21 | 4.43±0.28 | 12.28±2.51 | -9.2±0.72 | 58.74±0.64 |
| FAO standard | 6.5-8.4 | 3 | 13 | 1.25 | 50 |

**Note: EC:** electrical conductivity, **SAR:** sodium adsorption ratio, **RSC:** residual sodium carbonate

**Supplementary Table** **2.** Main characteristics of the biochar

| Parameter | pH | EC | OM | BD | Ash |
| --- | --- | --- | --- | --- | --- |
| Unit | - | dS m^-1^ | g kg^-1^ | Mg m^-3^ | % |
| Value | 7.75 | 2.84 | 428.5 | 0.299 | 46.78 |

**Note: EC:** electrical conductivity, **OM:** organic matter, **BD:** bulk density

**Supplementary Table** **3.** Summary of Wetland Units

| **Parameter** | Length (m) | Width (m) | Depth of Substrate (m) | Total Volume (m3) | Flow rate (m^3^ sec^-1^) | HRT (day) |
| --- | --- | --- | --- | --- | --- | --- |
| Value | 1.2 | 0.4 | 0.4 | 0.192 | 1.15 × 10^-6^ | 1 |

HRT: overall hydraulic retention time

**Supplementary Table** **4.** Concentrations of potentially toxic elements (PTE ) in the wastewater of Bahr Elbaqr drain

| Parameter | Unit | Mean±SD | FAO limit |
| --- | --- | --- | --- |
| Aluminum (Al) | mg L^-1^ | 0.476 | 5.00 |
| Antimony (Sb) | mg L^-1^ | <0.009 ^bdl^ | - |
| Arsenic (As) | mg L^-1^ | <0.002^bdl^ | 0.10 |
| Barium (Ba) | mg L^-1^ | 0.48 | - |
| Cadmium (Cd) | mg L^-1^ | 0.21 | 0.01 |
| Chromium (Cr) | mg L^-1^ | <0.002 ^bdl^ | 0.10 |
| Cobalt (Co) | mg L^-1^ | <0.003^bdl^ | 0.05 |
| Copper (Cu) | mg L^-1^ | 0.208 | 0.20 |
| Iron (Fe) | mg L^-1^ | 0.108 | 5.00 |
| Lead (Pb) | mg L^-1^ | <0.007 ^bdl^ | 5.00 |
| Manganese (Mn) | mg L^-1^ | 0.570 | 0.20 |
| Nickel (Ni) | mg L^-1^ | <0.004 ^bdl^ | 0.20 |
| Selenium (Se) | mg L^-1^ | <0.007 ^bdl^ | 0.02 |
| Tin (Sn) | mg L^-1^ | <0.006 ^bdl^ | - |
| Vanadium (V) | mg L^-1^ | <0.001 ^bdl^ | 0.10 |
| Zinc (Zn) | mg L^-1^ | 0.013 | 2.00 |

*bdl: below detection limit

| 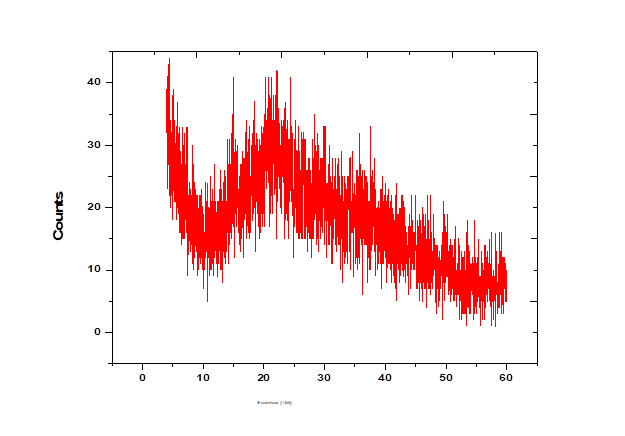  **A** | 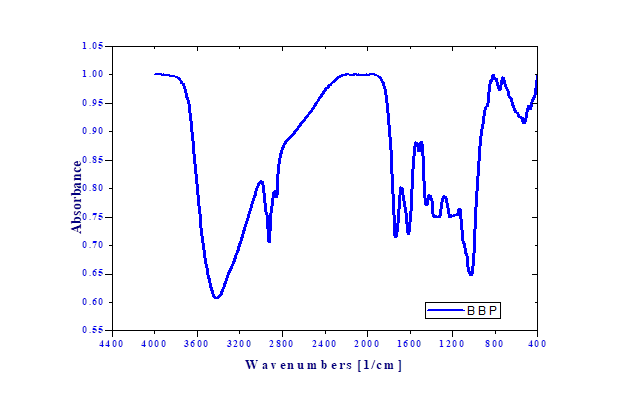  **B** |
| --- | --- |
| C  D   \| **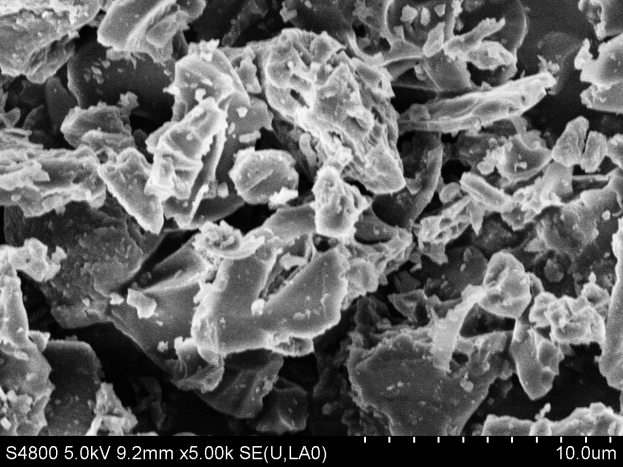** \| **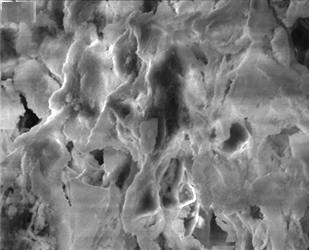** \| \| --- \| --- \| | |

Supplementary Fig. 1.  FTIR patterns (A), scanning electron microscopy (SEM) (B) of biochar before (C) and after (D) exposure to wastewater for 24 h.
